# Controversies on the Cusp: Anomalous Origin of the Left Coronary Artery From the Non-Coronary Cusp

**DOI:** 10.7759/cureus.7993

**Published:** 2020-05-06

**Authors:** Katia Bravo-Jaimes, Prakash Balan, Enrique Garcia-Sayan

**Affiliations:** 1 Division of Cardiovascular Medicine, University of Texas Health Science Center at Houston, Houston, USA

**Keywords:** coronary vessel anomaly, chest pain, computed tomography

## Abstract

Anomalous origin of the left coronary artery from the non-coronary cusp (LCANCC) is extremely rare and its prognosis and management are still controversial. We present two cases of symptomatic women with LCANCC and a comprehensive review of 19 studies reporting the prevalence, presentation, and management of LCANCC among 174,262 patients. Despite case reports of LCANCC in the pediatric population suggest a much worse prognosis, the optimal risk-stratification scheme for this type of anomaly in adults is yet to be defined, and it should not necessarily be considered a benign condition solely based on its anatomic origin or lack of an interarterial course.

## Introduction

The anomalous aortic origin of the coronary arteries (AAOCA) occurs in up to 0.7% of the general population [1]. Despite its extremely low prevalence, it is the second most common cardiovascular cause of sudden cardiac death (SCD) in competitive athletes. Those with an anomalous left coronary artery (LCA) arising from the right coronary cusp are at higher risk for myocardial ischemia and SCD, especially if factors such as slit-like ostium or an interarterial or intramural course are present [1]. Conversely, patients with anomalous right coronary artery arising from the left coronary cusp may have a more benign prognosis, especially if found in late adulthood [1]. To date, there are scarce reports of LCA arising from the non-coronary cusp (LCANCC), which is one of the rarest forms of coronary anomalies. Furthermore, the true prevalence, prognosis, risk stratification strategies, and management options for this specific coronary anomaly have not been well-defined. This case series describes two patients with LCANCC, with different clinical presentations, and reviews English-language studies in MEDLINE and EMBASE-indexed journals published from January 1968 to April 2019.

## Case presentation

Case 1

Patient Presentation

A 54-year-old woman presented for evaluation after multiple emergency room (ER) visits with atypical chest pain over the last three years. She denied dyspnea on exertion or syncope.

Diagnostic Workup

Troponins were repeatedly normal. An exercise 99m Tc-sestamibi myocardial perfusion single-photon emission computed tomography (SPECT) demonstrated normal perfusion, absence of scar, and no ST-segment changes or arrhythmias at a workload of 8.3 METs and 98% of the maximal predicted heart rate. A transthoracic echocardiogram (TTE) showed normal biventricular size and function and no valvular abnormalities.

**Figure 1:**
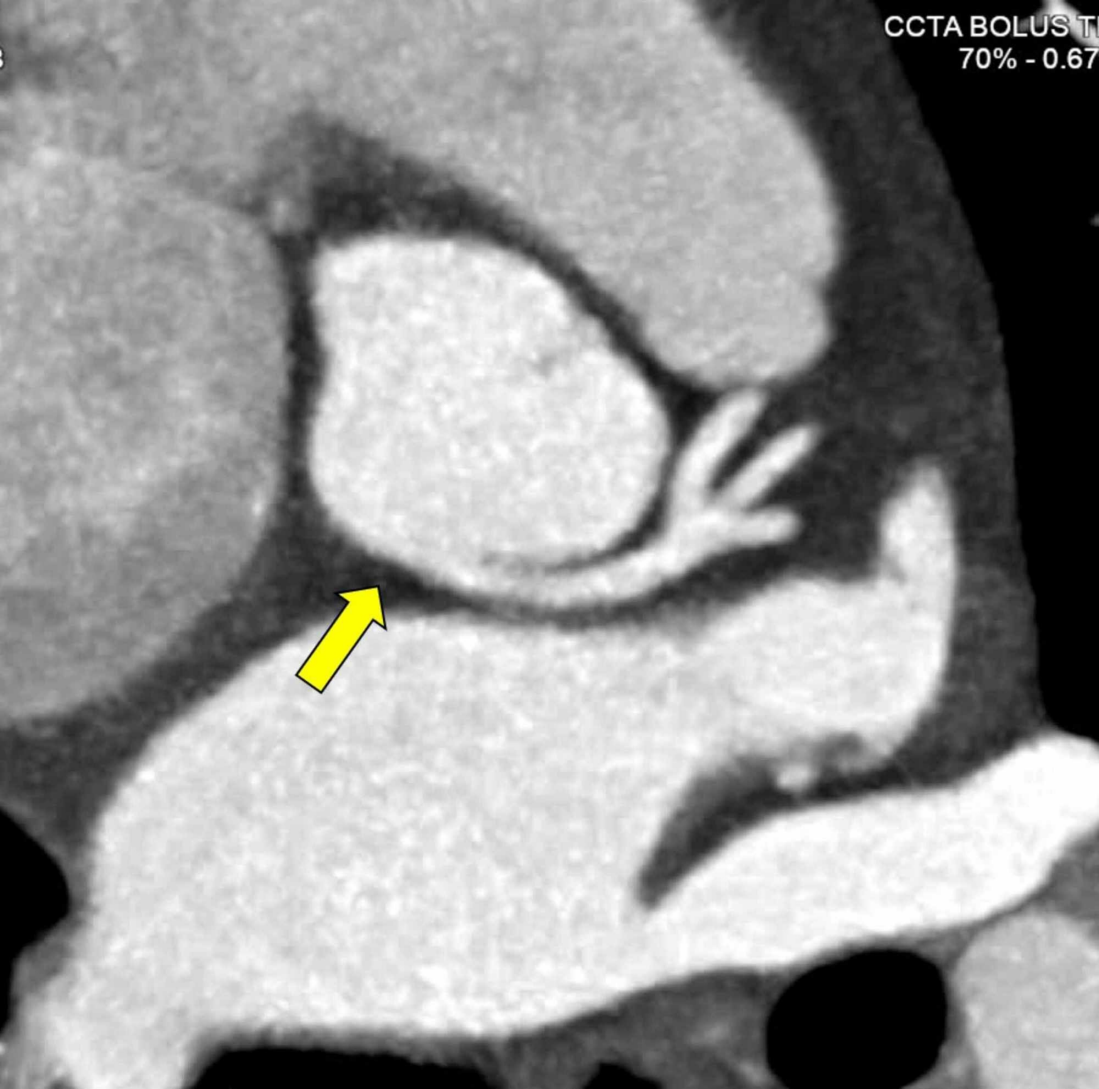
CCTA demonstrating LCANCC LCA with a retro-aortic course (arrow), trifurcating into the left anterior descending, ramus intermedius, and left circumflex arteries. CCTA: computed tomography coronary angiogram; LCANCC: left coronary artery from the non-coronary cusp; LCA: left coronary artery

Due to persistent chest pain episodes, computed tomography coronary angiogram (CCTA) was performed. The LCA had an anomalous origin from the non-coronary sinus of Valsalva followed a retro-aortic, extramural course and then trifurcated into the left anterior descending (LAD), ramus intermedius, and left circumflex arteries (LCX) (Figure 1). The right coronary artery (RCA) was dominant and originated from the right coronary cusp. There was no evidence of coronary atherosclerosis or myocardial bridging.

Interventions

No interventions were performed.

Outcomes

No antianginal medications were prescribed, and she has been uneventfully followed for 24 months without restrictions in her physical activities. During this time, she has not had episodes of chest pain requiring ER visits.

Case 2

Patient Presentation

A 54-year-old woman with morbid obesity, systemic lupus erythematous, hypertension, type 2 diabetes mellitus, chronic obstructive lung disease on home oxygen, and hyperlipidemia presented for evaluation after multiple ER visits with atypical chest pain over the last five years. She endorsed dyspnea on exertion but denied syncope.

Diagnostic Workup

Cardiac enzymes were repeatedly normal. SPECT using regadenoson demonstrated a large size, moderately severe anterior perfusion defect consistent with non-transmural ischemia. A TTE showed normal biventricular size and function, focal basal hypertrophy, and no valvular abnormalities.

**Figure 2:**
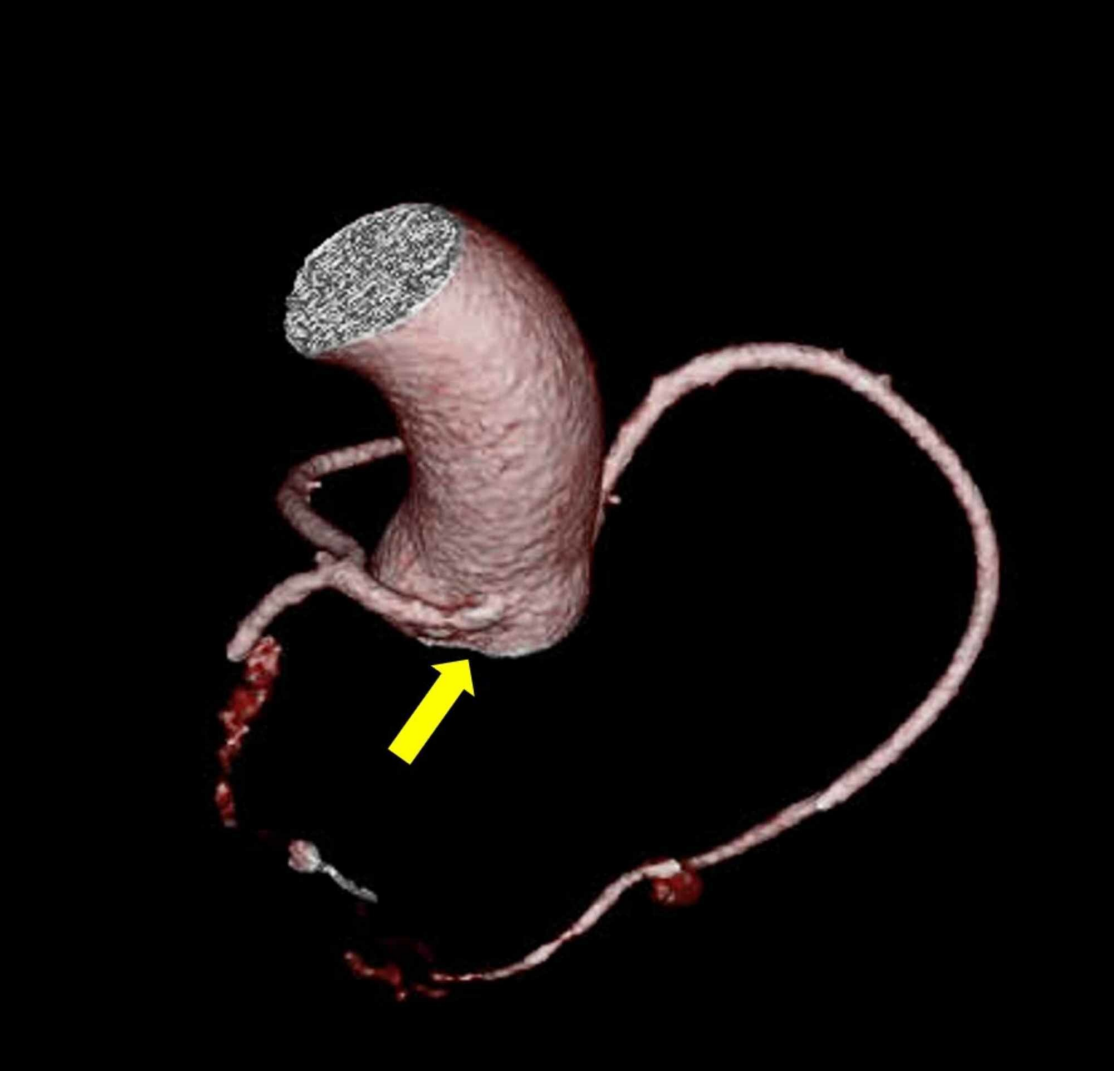
Tridimensional CT reconstruction showing LCANCC Tridimensional computed tomography reconstruction with leftward rotation showing the posterior origin of the LCA (arrow). CC: computed tomography; LCANCC: left coronary artery from the non-coronary cusp; LCA: left coronary artery

A CCTA showed that the LCA had an anomalous origin from the non-coronary sinus of Valsalva, followed a retro-aortic, extramural course, and then bifurcated into LAD and LCX (Figure 2). The RCA was dominant and originated from the right coronary cusp. The coronary artery calcium score was 0 Agatston units and there was no evidence of coronary stenosis or myocardial bridging.

Interventions

**Figure 3:**
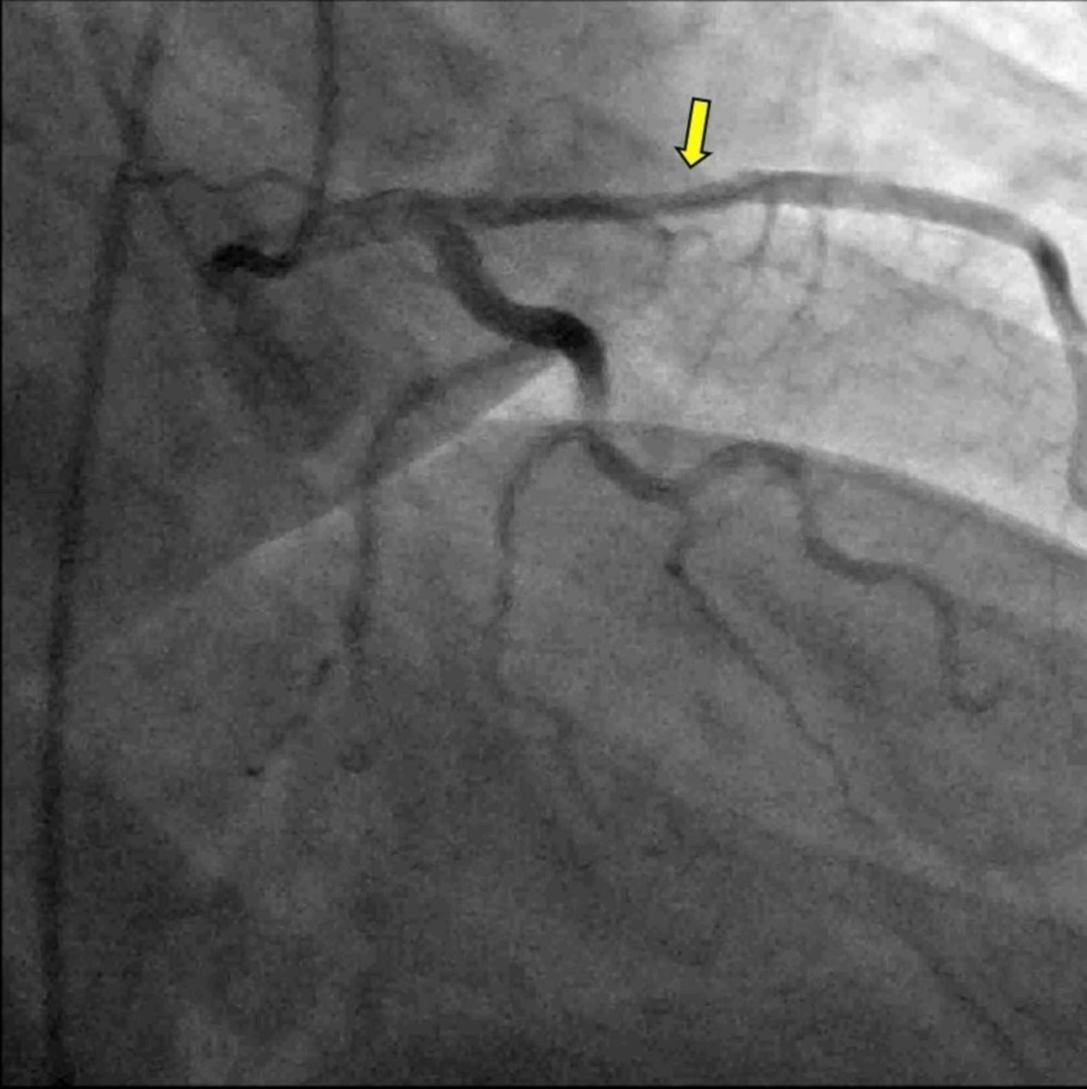
Coronary angiography in our second patient This study demonstrates 40% mid LAD stenosis (arrow) LAD: left anterior descending

**Figure 4:**
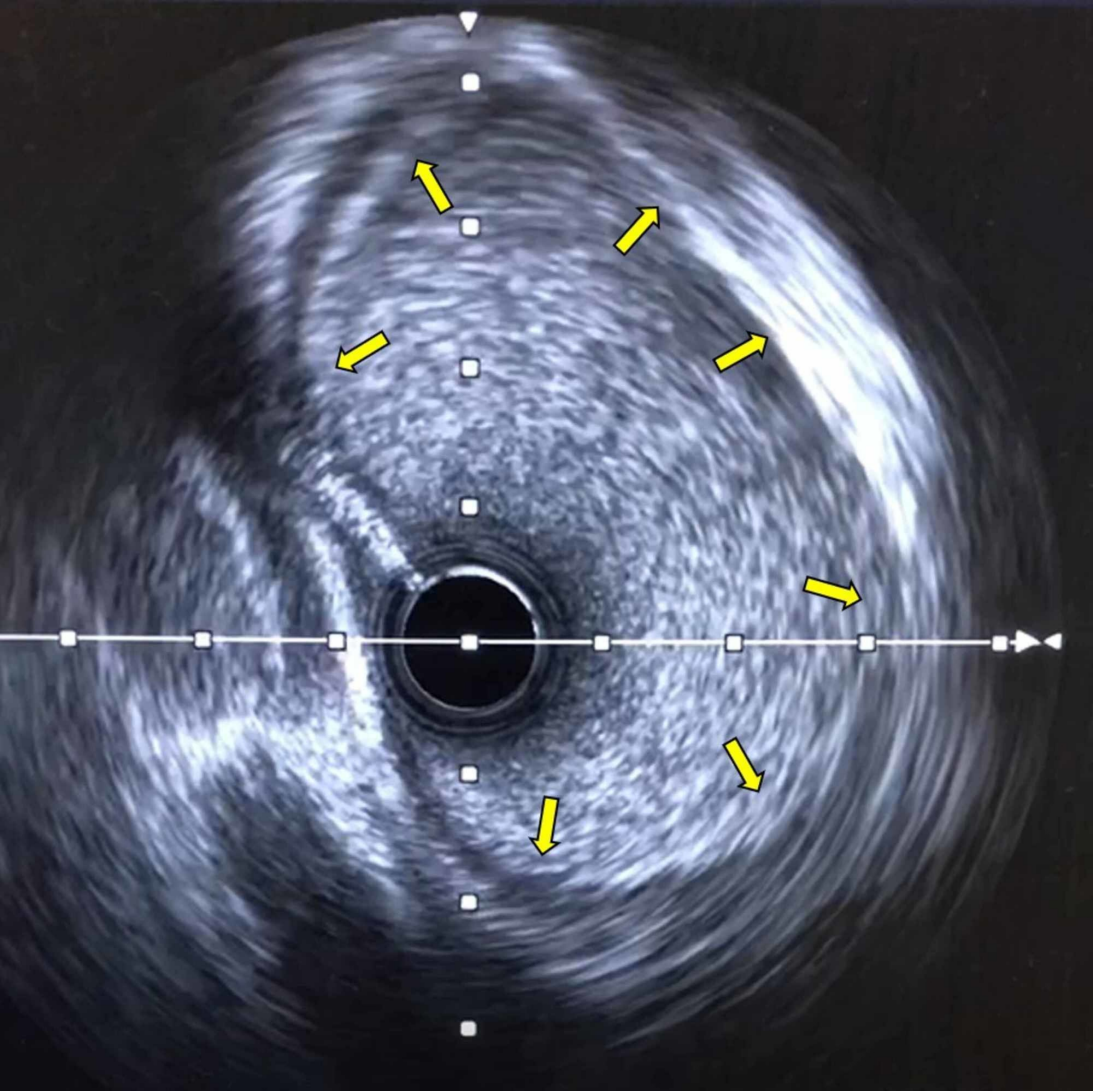
IVUS in our second patient IVUS at the level of LCA showing a lack of intramurality and no evidence of stenosis. IVUS: intravascular ultrasound; LCA: left coronary artery

Due to non-invasive evidence for myocardial ischemia, left heart catheterization and selective coronary angiography were performed. These revealed an elevated end-diastolic pressure at 23 mmHg, and a 40% mid LAD stenosis (Figure 3, Video 1). Fractional flow reserve (FFR) after adenosine infusion was calculated at 0.92 in the distal LAD. Intravascular ultrasound (IVUS) showed a left main coronary artery diameter of 6 mm and a minimal luminal area of 16 mm^2^. After the administration of dobutamine, the FFR was 0.9 and the minimal luminal area on IVUS was 16 mm^2^, respectively (Figure 4). Based on these findings, coronary intervention was not performed.

**Video 1:** LCA in right anterior oblique caudal view LCA: left coronary artery

Outcomes

Isosorbide mononitrate was started, and she has been uneventfully followed for 12 months without restrictions in her physical activities. During this time, she has not had episodes of chest pain requiring ER visits.

## Discussion

AAOCA has been associated with SCD, especially during exertion. Potential mechanisms explaining this association include (1) kinking or compression of the anomalous vessel (between the engorged aorta and pulmonary artery due to exertion-induced increased flow or due to an acute angle takeoff and resultant slit-like orifice with an insufficient coronary flow); (2) intermittent ischemia leading to myocardial fibrosis and subsequent foci for lethal ventricular arrhythmias; (3) intussusception; (4) coronary hypoplasia; (5) lateral compression of the coronary wall by the aorta; and (6) restriction of flow through the relatively noncompliant “pericommissural” area [1]. The prior high-risk findings (intramural, slit-like takeoff, or interarterial course) have emerged mostly from studies involving anomalous left or right coronary arteries arising from the opposite sinus of Valsalva, and to date, a comprehensive review of the data pertaining LCANCC is lacking.

We found a total of 19 studies reporting cases of LCANCC. A total of 36 out of 174,262 cases (1 in 4840 or 0.02%) were described, mostly from pathological reports (Table 1). Eighteen subjects (50%) were symptomatic, including 11 (31%) with sudden cardiac death (9 found on autopsy and 2 with exertional ventricular tachycardia), three (8%) with myocardial infarction (two involving the left coronary artery and another with triple vessel disease), and five (14%) with angina (3 cases did not report the management strategy or follow-up information and in 1 case, the patient had concomitant aortic coarctation). Nine (26%) patients had high-risk findings, such as a slit-like orifice (3), acute angle takeoff (5), and a proximal intramural segment (2). Only four (11%) patients had surgery: one with unroofing of the left coronary artery concomitantly with aortic coarctation repair, other with the marsupialization of the anomalous LCA, the third with patch enlargement of the proximal left coronary artery, and the last with triple vessel coronary bypass.

**Table 1:** Studies reporting patients with LCANCC * Included anomalous origin of the right and/or left coronary arteries from the noncoronary cusp as a group LCANCC: left coronary artery from the non-coronary cusp; SCD: sudden cardiac death, SIHD: stable ischemic heart disease, CASS: coronary artery surgery study, STEMI: ST-elevation myocardial infarction, MRI: magnetic resonance imaging, CT: computed tomography, TEE: transesophageal echocardiogram, ICD: implantable cardiac defibrillator

First author, Year	Number of patients with LCANCC	Population	Clinical Presentation	Diagnostic modality	High-risk findings/Treatment
Taylor et al., 1992 [2]	17/242*	US, military, children and adults with congenital coronary anomalies	5/17 SCD	Pathology	--
Ogden et al., 1968 [3]	1/244	US, congenital coronary anomalies	--	Pathology	--
Click et al., 1989 [4]	1/24959	US and Canada, adults with SIHD undergoing coronary angiography (CASS study)	--	Angiography	--
Yamanaka et al., 1990 [5]	1/126595	US, adults undergoing angiography	Asymptomatic	Angiography	--
Ishikawa et al., 1990 [6]	1	Japan, 12-year-old girl	Anterolateral STEMI	Pathology	Slit-like orifice/--
Cohen et al., 1991 [7]	1	US, 68-year-old female	Angina	Angiography	--
Kaku et al., 1994 [8]	1	Japan, 47-year-old male	Angina	Angiography, MRI, TEE	Acute angle takeoff/--
Frescura et al., 1998 [9]	1/1200	Italy, children and adults with isolated coronary anomalies	Perinatal asphyxia	Pathology	--
Garg et al., 2000 [10]	1	US, 62-year-old male	Angina	Angiography, CT	--
Hamamichi et.al., 2000 [11]	1	Japan, 12-year-old girl	SCD, myocardial infarction	Pathology	Slit-like orifice, acute angle takeoff/--
Mavi et al., 2004 [12]	1/10042	Turkey, adults undergoing coronary angiography	Anterior STEMI	Angiography	Triple vessel coronary bypass
Liberman et al., 2005 [13]	1	US, 11-year-old boy	Aborted SCD	TTE, MRI, angiography	Acute angle takeoff/ICD
Catanzaro et. al., 2005 [14]	1	US, 16-year-old male	SCD	Pathology	Acute angle takeoff/--
Alphonso et. al., 2007 [15]	1	US, 11-year-old girl	Aborted SCD	Angiography	Slit-like orifice/Patch enlargement proximal LCA
Fujimoto et al., 2011 [16]	Feb-69	Japan, adults undergoing coronary CT	--	CT	--
Nishiyama et. al., 2011 [17]	1	Japan, 13-year-old girl	Aborted SCD	CT	Acute angle takeoff
Ganga et al., 2018 [18]	1	India, 54-year-old male	Angina, coarctation of the aorta	CT	Intramural/Unroofing
Finocchiaro et al., 2019 [19]	Jan-00	UK, children and adults with SCD as the cause of death	SCD	Pathology	Intramural/--
Larmeu et al., 2019 [20]	1	Netherlands, 15-year-old girl	Angina, syncope	CT	--/Marsupialization

Due to the rarity of this condition, clear pathological associations are difficult to identify. The study by Taylor et al. had the highest number of cases of SCD in patients with LCANCC, however, it did not specify the frequency of high-risk findings, clearly limiting the observation that this specific coronary anomaly may inherently carry a higher risk of SCD [2]. Furthermore, nine out of the 17 subjects who experienced SCD, angina, or ST-elevation myocardial infarction had high-risk features, arguing in favor that high-risk features determine overall prognosis rather than anatomic origin.

The absence of high-risk anatomic findings, as well as the normal exercise myocardial perfusion imaging in our first patient, argued against surgical management. It is important to remark though that some patients with AAOCA may have intermittent ischemia with false-negative stress test results, which makes risk-stratification with routine stress testing difficult. This patient has been uneventfully followed for at least two years without restrictions in her physical activities.

In our second patient, extensive evaluation, including CCTA, intravascular ultrasound, and FFR did not reveal intramurality or luminal stenosis at rest and after the administration of dobutamine. From prior reports, vasodilators do not mimic exercise-induced great vessel engorgement, however, in the absence of an interarterial course, this may not be a determinant. Whether or not a slit-like orifice in the absence of a reduced minimal luminal area or diameter confers a higher risk of SCD or myocardial infarction needs to be better elucidated in further studies.

## Conclusions

We report two cases of women with LCANCC, with different clinical presentations and no high-risk features who were managed conservatively with favorable results, and reviewed the current body of evidence addressing this rare coronary anomaly. Since prior case reports of LCANCC in the pediatric population suggest a much worse prognosis, the optimal risk-stratification scheme for this type of anomaly in adults is yet to be defined, and it should not necessarily be considered a benign condition solely based on its anatomic origin or lack of interarterial course.
